# ENHANCEFITNESS FOR ADULTS WITH MEMORY LOSS AND CARE PARTNERS: A PILOT

**DOI:** 10.1093/geroni/igae098.0999

**Published:** 2024-12-31

**Authors:** Basia Belza, Zih-Ling Wang, Sarah McKiddy, Kelly Ash, Paige Denison, Judi Pirone, Meghan Thompson, Allison Webel

**Affiliations:** University of Washington, Seattle, Washington, United States; University of Washington, Seattle, Washington, United States; University of Washington, Seattle, Washington, United States; Northshore Senior Center, Bothell, Washington, United States; Sound Generations, Seattle, Washington, United States; Northshore Senior Center, Bothell, Washington, United States; Sound Generations, Seattle, Washington, United States; University of Washington, Seattle, Washington, United States

## Abstract

The purpose of this pilot was to evaluate the feasibility of an in-person evidence-based, physical activity intervention, Enhance®Fitness (EF), for community-dwelling adults with memory loss and care partners. In partnership with a senior center in the Pacific Northwest we recruited dyads to participate in a one-hour EF class, three times per week for eight weeks. Each dyad consisted of one participant with memory loss and their care partner. We assessed depression, cognition, and strength and agility at baseline and post-intervention. We enrolled 8 dyads/16 participants. Most (n=6) care partners were spouses. At baseline, 5 participants had normal cognition and 5 mild, 2 moderate, and 4 had severe cognitive impairment. Eight participants were below average in strength and 12 participants below average on agility. Six of 8 dyads dropped out prior to conclusion of the program due to injury before the start of the study or illness and fatigue during the study. Two dyads who completed the eight-week program attended, on average, 19 of 21 classes. After the intervention of those dyads who completed the program there was improved cognition and strength but worse depression scores. In an exit interview participants reported liking EF for its group setting, focus on physical activity, social interactions, and relationship building. They appreciated that the instructor maintained an engaging and personalized experience and requested more visual and verbal cues. Care partners wanted an assistant to help their loved ones with exercises. Additional adaptations and support are needed for this population to participate safely in EF.

